# Acupuncture for hiccups: A systematic review protocol of high-quality randomized trials

**DOI:** 10.1097/MD.0000000000018343

**Published:** 2019-12-20

**Authors:** Xiao-Bing Li, Dong-Jie Wu, Min-Chun Yang

**Affiliations:** a Department of Gastroenterology, The First People's Hospital of Pinghu City, Jiaxing; b Department of Chinese Medicine, Zhejiang Hospital, Hangzhou, China.

**Keywords:** acupuncture, hiccups, systematic review

## Abstract

**Background:**

A Hiccup is a common disease that often occurs along with other chronic or acute conditions. At present, there is a lack of feasible therapies for hiccups, and acupuncture is a treatment method with enormous clinical practice worldwide.

**Methods:**

Based on a pre-defined search strategy, we searched seven databases and screened them by two independent investigators, without language and publication status restriction from inception to date. We use the pre-set form to incorporate data and utilize Revman software to synthesize data. We will evaluate the risk of bias in the inclusion of the study based on the Cochrane ‘Risk of bias’ assessment tool. The quality of the evidence will be evaluated according to the GRADEpro software.

**Results:**

This systematic review will evaluate the efficacy and safety of acupuncture treatment for hiccups. The entire process will be referred to the Cochrane handbook recommended by the Cochrane Collaboration.

**Conclusion:**

This review will provide systematic evidence to summarize whether acupuncture is an effective intervention in the treatment of hiccup.

**Key points:**

Strengths and limitations of this study.
The first systematic review of the efficacy and safety of acupuncture treatment of hiccups, while the hiccups intervention is extremely lacking.
Included studies were screened and data extracted by 2 independent authors.
The quality of the literature is limited because of poor design.

## 1 Introduction

### 1.1 Description of the condition

A hiccup is a contraction movement caused by diaphragmatic spasm, a short sound that suddenly closes the glottis when inhaling.^[1,2]^ This is a disease that can heal itself, but stubborn hiccups can seriously affect the life quality of patients which are difficult to cure.^[3,4]^ A retrospective study found that 55 out of every 100,000 patients in that hospital were initially diagnosed as having a hiccup.

Hiccups are divided into 3 types due to duration: acute, persistent, and intractable hiccups.^[5,6]^ Acute hiccups do not exceed 48 hours, are common in children and sometimes occur in adults. The duration of persistent hiccups ranges from 2 days to a month, and intractable hiccups last more than 1 month.^[7]^ The duration of persistent hiccups lasts more than 2 days, and the intractable hiccup lasts more than a month. Pharmacological treatments are limited.^[8]^ Based on limited evidence of efficacy and safety, baclofen and gabapentin may be first-line therapy for the intractable hiccups and persistent hiccups.^[9–11]^ Recommended on metoclopramide and chlorpromazine are in reserve.

### 1.2 Description of the intervention

Acupuncture therapy is based on the theory of Traditional Chinese medicine.^[12–14]^ Acupuncturists inserted needle into the patients’ skin according to a certain angle to coordinate body functions.^[15]^ The acupuncture manipulation such as twisting and lifting is used to stimulate specific parts of the body to achieve the purpose of curation. The point needles aim to insert into is called the acupoint.^[16,17]^ According to the latest acupuncture textbook statistics, the human body has a total of 361 meridian acupoints.^[18–20]^

The types of acupuncture treatments included: body acupuncture, scalp acupuncture, auricular acupuncture, electroacupuncture, and acupressure.^[21,22]^

The control group included placebo, sham acupuncture, conventional therapies, or other treatments.

### 1.3 How the intervention might work

Stimulation performed by a clinician using steel needle for acupuncture points produces a neuromodulation-like regulation. Some experiments and clinical trials have found that stimulating certain acupoints produce the effect of inhibiting nausea and vomiting.^[23–25]^ Lin reckoned that the therapeutic effects of acupuncture are produced by activating high-threshold mechanical receptors that stimulate tissue.^[23]^

### 1.4 Why it is important to do this review objective

A hiccup is a common disease, but its mechanism is still not clear.^[26–28]^ The treatment of hiccups is very limited and there are few randomized clinical trials emerged. Massive clinical studies on acupuncture treatment for hiccups came up, although the quality of evidence is unsatisfactory. Previously, a Cochrane review had included acupuncture intervention for hiccups, but they did not conduct quantitative analysis due to the quality and quantity of the literature.^[29]^ Our systematic review brings current evidence of acupuncture for hiccups.

### 1.5 Objective

To assess the effectiveness and safety of acupuncture for hiccups.

## 2 Methods

### 2.1 Types of studies

Inclusion criteria:

- Truly randomized controlled trials including double blinded, single-blinded and cluster-randomized trials published in any language.

Exclusion criteria:

- Quasi-randomized trials.

### 2.2 Types of participants

The patient was diagnosed as hiccups by a clear diagnosis. We will record the diagnostic criteria referenced by the authors.

### 2.3 Types of interventions

For therapeutic and preventive objective, acupuncturists insert the needles to participant's skin at several fixed positions, which are called acupoints.

Randomized control trials of acupuncture and similar therapies for hiccups will be included. Quasi-random means that the methods of allocating patients are not strictly random such as the patient's birth date or hospital record number will be excluded. There is no language restriction about language.

### 2.4 Types of outcome measures

#### 2.4.1 Primary outcomes

1. Cessation of hiccups within a specified time period following intervention.
2. Any change in hiccups frequency, or subjective or objective change in hiccups intensity.
3. Adverse events.

#### 2.4.2 Secondary outcomes

1. Minor adverse events not requiring withdrawal of intervention.

### 2.5 Search strategy

In order to collect RCTs as much as possible, we will search the following databases and develop a search strategy.

The Cochrane Central Register of Controlled Trials (CENTRAL) (*The Cochrane Library*, Issue 3, 2019);
MEDLINE (1950–2019);
EMBASE (1980–2019);
AMED (Allied and Complementary Medicine) 1985–2019;
CBMdisc (1999–2019);
CNKI (1999–2019);
VIP (1999–2019).

The search strategy will be recorded in Table 1.

**Table 1:**
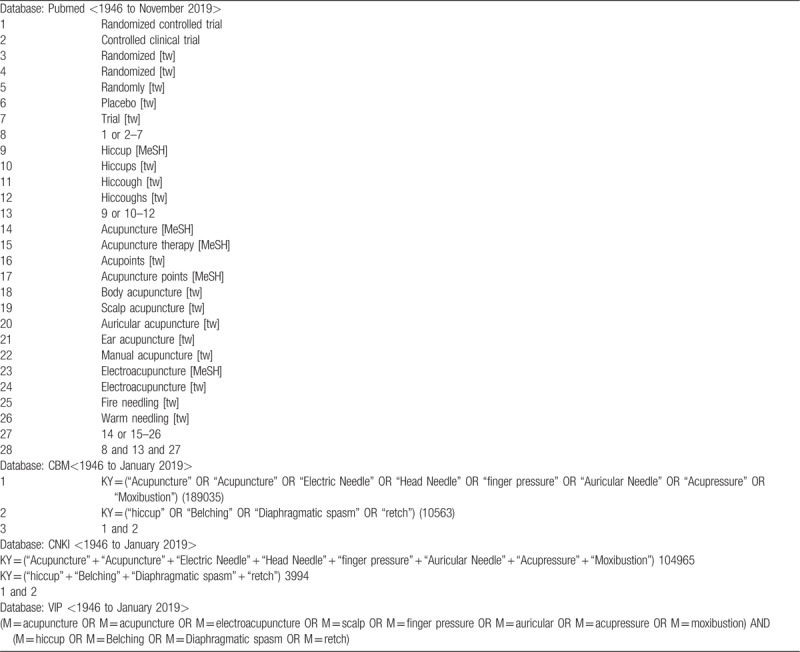
Search strategy of acupuncture for hiccup.

### 2.6 Searching other resources

We will manually search for other related journals and conference abstracts. We will also search for literature based on references included in the study.

### 2.7 Data collection and analysis

#### 2.7.1 Selection of studies

Two investigators independently read the topics and abstracts to identify the literature that might be included.

**Figure 1:**
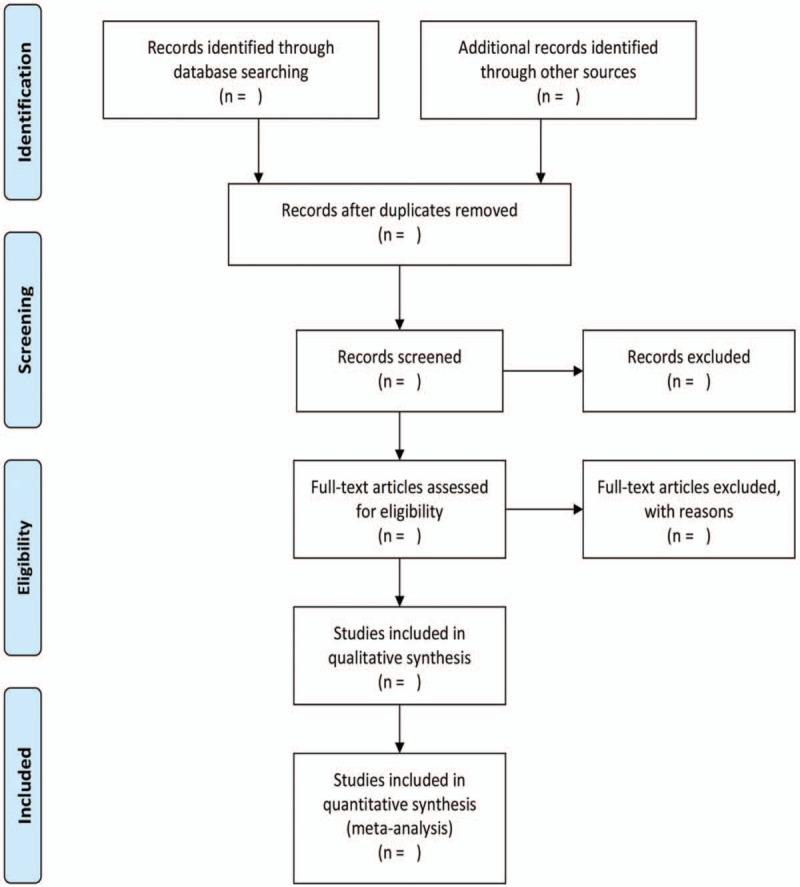
Literature screening flow diagram has 4 processes: Identification, Screening, Eligibility, and Included. Literature are collected from database and other resources, and literatures that do not meet the inclusion criteria is excluded. Final literatures are included according to eligibility criteria.

The authors will read the full text independently to determine if the study meets the inclusion criteria. Inconsistencies in the inclusion process are determined by the third author. If the data in the literature is not sufficient, we will contact the author of the article to ask for missing data. The specific inclusion process will refer to the “PRISMA” flow chart (Fig. 1).

#### 2.7.2 Data extraction and management

The 2 researchers will use the pre-designed data extraction form to extract the data. We include data from several categories, including Methods, Patients, Interventions, Outcome, and other Indicators. We will build table “Characters of included study” to organize the feature of studies. We will discuss to resolve the inconsistencies. One author enters the data into the Review Manager Version 5.3 (Cochrane Collaboration, Oxford, UK), while another author check the data.

#### 2.7.3 Assessment of risk of bias in included studies

We will evaluate the risk of bias in the inclusion of the study based on the Cochrane “Risk of bias” assessment tool.^[30]^ The domains that need to be evaluated include: selection bias (random sequence generation and allocation concealment); performance bias (blinding of participants and personnel); detection bias (blinding of outcome assessment); attrition bias (completeness of outcome data); reporting bias (selective reporting) and other potential sources of bias. The authors will evaluate the risks of these inclusion studies, and the inconsistencies are determined by the third author. We grade the risk of bias into low, unclear, and high.

All biases of included study will be presented in table.

#### 2.7.4 Measures of treatment effect

We will conduct data analysis based on the Cochrane guidelines recommended by The Cochrane Collaboration.^[30]^ For continuous variables, mean difference (MD) or standardized mean difference (SMD) and 95% confidence interval (CI) will be used to pool the data. For dichotomous variables, odds ratios (OR) and 95% CI will be used to pool the data. Review Manager Version 5.3 software will be used to analyze and synthesize the data for this study.

#### 2.7.5 Unit of analysis issues

When the treatment time for different studies of continuous variables is inconsistent, we will only include the data from the first phase.

#### 2.7.6 Dealing with missing data

We will analyze data on an intention-to-treat basis where possible. We will contact primary study authors by phone or email to obtain any missing patient data. We will record the rate of contact. We will note discrepancies between the number of patients enrolled and number of patients in whom outcomes were reported in the “Characteristics of included studies” table. For other outcomes, we will only analyze the available data.

#### 2.7.7 Assessment of heterogeneity

We intend to use the concealment of allocation grading in the investigation of any heterogeneity and in sensitivity analyses. Other aspects of study quality include the extent of blinding (if appropriate), the extent of losses to follow-up, non-compliance, whether the outcome assessment was standardized and whether an intention-to-treat analysis was undertaken. We will identify heterogeneity by visual inspection of the forest plots and by using a standard Chi^2^ test with an alpha significance level of 0.1. We will specifically examine heterogeneity (variation) between the results of different studies using the *I*^2^ statistic. An *I*^2^ value ranging from 50% to 90% may indicate substantial heterogeneity.^[30]^ We intend to perform pre-determined sub-analyses in order to explore the cause(s) of heterogeneity. Assessment of reporting biases in view of the difficulty in detecting and correcting for publication bias and other reporting biases, we will aim to minimize their potential impact by ensuring a comprehensive search for eligible studies and by being alert for duplication of data. If there are 10 or more studies in an analysis, we will use a funnel plot to explore the possibility of small study effects (a tendency for estimates of the intervention effect to be more beneficial in smaller studies).

#### 2.7.8 Data synthesis

If the studies are sufficiently similar, we will combine the data from primary studies using a fixed-effect model in the following comparisons:

1. Acupuncture versus placebo;
2. Acupuncture versus conventional treatment.
3. Acupuncture plus versus placebo;
4. Acupuncture plus versus conventional treatment.

We will display graphically an increase or decrease in the odds of a particular outcome, which may be beneficial or detrimental, in the meta-analyses. We will display an increase in the odds of an outcome to the right of the centre line and a decrease in the odds of an outcome to the left of the centre line. In the event of substantial clinical, methodological, or statistical heterogeneity, we will not combine study results by means of meta-analysis, but instead will summarize them in narrative form: subgroup analysis and investigation of heterogeneity. Where data are available, we will conduct subgroup analyses to determine the evidence for acupuncture in different subsets of subfertility and for different outcomes, according to the different types of acupuncture, in order to investigate heterogeneous results.

#### 2.7.9 Sensitivity analysis

We will undertake sensitivity analysis to examine the stability of the results in relation to a number of factors relating to the way a study was done. We will repeat the analysis as follows:

1. Excluding studies based on the “Risk of bias” assessment, for example, first low risk of bias for allocation and then high or unclear risk of bias for allocation (in other words excluding studies without adequate safeguards for allocation concealment) (quasi-randomized studies will be excluded);
2. Excluding studies of poor overall methodological quality; we will consider the following aspects of quality for this sensitivity analysis: inadequate blinding, no stated method of diagnosis, incomparable groups (either because they have different baseline characteristics or they do not have identical care programmes), no intention-to-treat analysis;
3. Using a random-effect model.

Overall quality of the body of evidence: “Summary of findings” table. We will generate a “Summary of findings” table using GRADE pro software. The table will evaluate the overall quality of the body of evidence for the main review outcomes, using GRADE criteria (study limitations (i.e. risk of bias), consistency of effect, imprecision, indirectness, and publication bias). We will justify, document and incorporate into our reporting of results for each outcome our judgements about evidence quality (high, moderate, or low).

#### 2.7.10 Subgroup analysis and investigation of heterogeneity

We will carry out subgroup analysis to detect the source of heterogeneity according to different interventions and outcome measures. The different type of hiccups (such as persistent and intractable hiccups) is an important factor to conduct subgroup analysis.

## 3 Discussion

Liu et al reported a 77-year-old patient who was diagnosed as hiccups which lasted more than 7 days after acute myocardial infarction. Experiencing routine therapy such as baclofen was declared invalid, acupuncture was used to stimulate the acupoint GV14 (Da zhui) to terminate the hiccups.

A retrospective case series was conducted at the Clinical Research Centre of American.^[31]^ They recruited 16 persistent hiccups patients with cancer to measure the treatment efficacy of acupuncture. According to the result, the study investigators conclude the acupuncture may be a low-cost, efficacy therapy for persistent hiccups patients with cancer.

## Author contributions

**Conceptualization:** Xiao-Bing Li, Min-Chun Yang.

**Supervision:** Min-Chun Yang.

**Writing – original draft:** Dong-Jie Wu.
